# Contactless In Situ Electrical Characterization Method of Printed Electronic Devices with Terahertz Spectroscopy

**DOI:** 10.3390/s19030444

**Published:** 2019-01-22

**Authors:** Mariia Zhuldybina, Xavier Ropagnol, Charles Trudeau, Martin Bolduc, Ricardo J. Zednik, François Blanchard

**Affiliations:** 1 Département de Génie Électrique, École de Technologie Supérieure (ÉTS), Montréal, QC H3C1K3, Canada; mariia.zhuldybina.1@ens.etsmtl.ca (M.Z.); ropagnol@emt.inrs.ca (X.R.); charles.trudeau.2@ens.etsmtl.ca (C.T.); mbolduc@varitron.com (M.B.); 2 Institut National de la Recherche Scientifique, Énergie, MatéRiaux et Télécommunications (INRS-EMT), Varennes, QC J3X1S2, Canada; 3 Département de Génie Mécanique, École de Technologie Supérieure (ÉTS), Montréal, QC H3C1K3, Canada; ricardo.zednik@etsmtl.ca

**Keywords:** printed electronics, inkjet printing, terahertz time-domain spectroscopy, vortex phase plate, vortex beam

## Abstract

Printed electronic devices are attracting significant interest due to their versatility and low cost; however, quality control during manufacturing is a significant challenge, preventing the widespread adoption of this promising technology. We show that terahertz (THz) radiation can be used for the in situ inspection of printed electronic devices, as confirmed through a comparison with conventional electrical conductivity methods. Our in situ method consists of printing a simple test pattern exhibiting a distinct signature in the THz range that enables the precise characterization of the static electrical conductivities of the printed ink. We demonstrate that contactless dual-wavelength THz spectroscopy analysis, which requires only a single THz measurement, is more precise and repeatable than the conventional four-point probe conductivity measurement method. Our results open the door to a simple strategy for performing contactless quality control in real time of printed electronic devices at any stage of its production line.

## 1. Introduction

Printable electronics (PE) is an advanced manufacturing technology that is of significant interest to a large range of industries, from consumer goods, electronics, aerospace, automotive, pharmaceutical, biomedical, to textiles and fashion [1,2,3,4,5]. It offers an attractive alternative to conventional circuit manufacturing by enabling lower-cost, maskless, and rapid production of customized electronic devices [6]. PE is compatible with a wide range of substrates, as long as they are not porous and can resist all fabrication steps, including pre- and post-printing processes [7]. In addition, various kinds of conductive, semi-conductive, and dielectric inks are now commercially available. Therefore, PE allows the realization of unique electronic components that can be bent, twisted and stretched, all while retaining their electrical properties [8,9,10,11]. In recent years, the development of various contact- and non-contact printing technologies, such as flexography, gravure, screen- or inkjet-printing, has advanced significantly [6]. Post-printing processes also play a key role in the manufacturing of PE devices. The most commonly used sintering approaches are conventional thermal annealing, electrical sintering, microwave, and photonic sintering by either continuous-wave laser irradiation or high-power flashing lamps [7,12]. While the spatial resolution and definition of the device are related to the printing method, the quality of the electrical properties of the printed devices is directly related to the post-printing process [12]. Particularly, the solid and uniform dielectric or metallic tracks from the printed pattern are obtained during this step.

The quality of PE devices can be evaluated using different types of microscopy, such as atomic force microscopy, scanning electron microscopy or optical microscopy [13,14], which are well-established tools for analyzing the surface morphology of materials. Nevertheless, these techniques are expensive, slow, and allow limited surface area observation. Other types of characterization techniques, such as crystallography analysis, thermography, elecro- or photo-luminescence, are also time-consuming and require special conditions, such as vacuum or helium environments, to avoid noise and damage [15,16,17,18]. The electrical conductivity of printed traces in flexible PE circuits is assessed using conventional methods drawn from the electronics industry, e.g., the flying probes or four-point probe method (4PP). However, these techniques cannot be envisioned for high-volume roll-to-roll (R2R) printing since in-line contact methods are not compatible with continuous manufacturing tools. Thus, a non-contact conductivity characterization method is necessary.

Traditional graphic art printing or off-set printing used in the manufacture of full-color magazines, posters, packaging, etc., evaluates print quality using a color control bar (GATF Standard Offset Color Bar) [19]. Using a densitometer or a spectrophotometer, these bars allow for accurate determination of ink density, dot gain, and screen angle accuracy. Generally, the color control bars are printed away from the immediate image area, and are often cut off or hidden during final assembly. Similarly, for PE production, an in situ quality control characterization technique has to be developed. Time-domain spectroscopy (TDS), using electromagnetic terahertz (THz) radiation, i.e., for frequencies ranging from 100 GHz to 10 THz, is a powerful tool that allows non-destructive characterization, and which is very sensitive to the conductivity of matter [20,21]. THz waves have previously been used to characterize carbon printed ink with the THz imaging method [22]. However, for high volume production, such approach is time consuming and may require complicated data analysis to efficiently recover the conductive property of the printed devices. Alternatively, THz engineered structures, such as metamaterials [23], can exhibit a strong response in transmission- or reflection-type geometries with a high dependency on material conductivity [24,25]. Therefore, it can provide a straightforward sensing tool to retrieve the conductive property of the printed ink. Already, THz metamaterials printed by inkjet [26,27,28,29], digital aerosol jet [30], laser printing [31] or electro-hydrodynamic jet [32,33,34] printing have been reported, allowing for rapid fabrication of THz metamaterial-based sensors and functional THz devices using PE methods [27,28,29,30,31,32,33,34].

**Figure 1:**
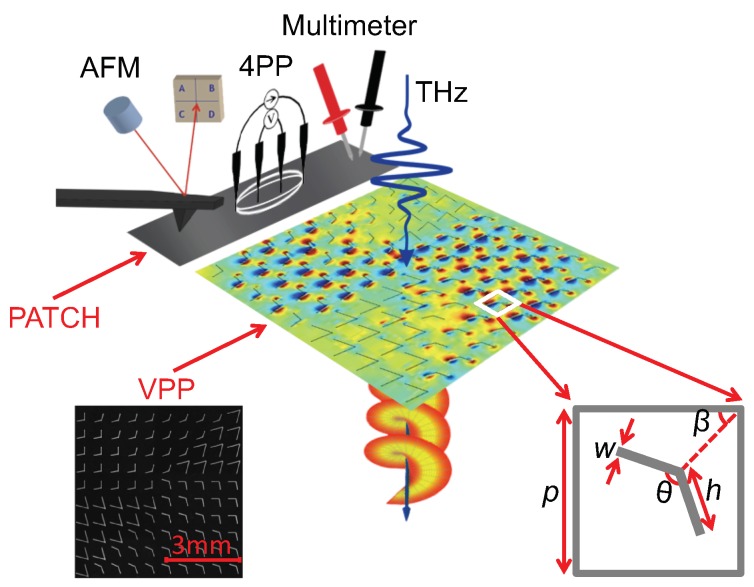
Sketch depicting the provided measurements: atomic force microscopy (AFM), four-point probe (4PP), multimeter, terahertz time-domain spectroscopy (THz-TDS). The left inset is the visible image of the center part of a printed vortex phase plate (VPP). The right inset shows one representative V-shaped antenna unit with the geometrical parameters. Dimensions of p=600 μm, w=30 μm, $\beta =45^{\circ }$ were kept the same for all antennas. The length of the slit h=234, 246, 270, 450 μm and the angle between slits $\theta =130^{\circ }$, $120^{\circ }$, $100^{\circ }$, $60^{\circ }$ according to order of antennas in [37].

In this work, a THz engineered resonance structure has been developed as a quality control bar to probe the post-printing manufacturing process of PE devices. Our objectives were to determine the transmission resonant behavior of a control bar using THz waves as a function of ink conductivity and to link the THz frequency conductivity with the static conductivity of printed devices that are manufactured simultaneously (i.e., with the same sintering condition). As illustrated in Figure 1, we have performed a comparative study between THz inspection of a resonant printed structure against conventional conductivity measurement methods on a printed structure, i.e., using multimeter (MM), four-point probe (4PP) and atomic force microscopy (AFM). Our THz measurements are well-correlated with the non-resonant printed structure conductivities and confirm the ability to determine the quality of the post-printing manufacturing process of PE devices by THz inspection of a simple control bar showing a distinctive response in the THz frequency range. To retrieve the resonance response of our control bar, standard terahertz time-domain spectroscopy (THz-TDS) was utilized. In addition, the well-known THz transmission method was compared through a novel dual-wavelength THz spectroscopy (DWTS) analysis. We show that DWTS determines the conductivity of the PE device using a single scan measurement. Additionally, our method does not rely on THz phase-sensitive measurements, and is therefore ideally suited for next-generation low-cost THz emitters and sensors [35,36] and opens the door to contactless in situ quality control of PE devices.

## 2. Materials and Methods

### 2.1. Sample Fabrication

We designed a special printed pattern sample consisting of two parts: (i) a resonant structure at THz frequency, and (ii) a rectangular “patch” sample. These two patterns will serve as comparative tools between THz spectroscopy and conventional methods, respectively. As shown in Figure 1, the resonant “control bar” consists of a THz vortex phase plate (VPP) made of V-shape antennas [37], whereas the “patch” consists of a 1×10 mm${}^{2}$ printed rectangular shape.

The unit cell design of the VPP antenna yields a specific resonant response to electromagnetic waves, and as commonly known for metamaterial structures, these properties are preserved in a macroscopic medium fabricated from their individual units. Similarly, as for electrically tunable metamaterials, here the variability in resonance response was probed as a function of ink conductivity. As expected for metamaterials, a printed VPP sample with lower conductivity will cause the resonance to be damped [38].

The VPP with topological number l=1 was designed according to the work of Jignwen He et al. [37]. It is made of eight sectors, which supply a phase changing from 0 to 2π with a step size of π/4. Each sector was formed from one type of V-shaped antenna, as depicted in the right inset of Figure 1, and made from two rectangular slits attached at one end at a specific angle (θ). Similarly, like [37], we kept all geometrical aspect values of angles θ and β, whereas β was the angle between the bisector line of a V-shaped antenna and the *x*-axis. Due to the resolution of our printer, and according to the frequency spectrum of our THz source, the dimensions of the unit cell (*p*) and the length of the slit (*h*) were increased three-fold. A feature width (*w*) of 30 μm was set and chosen according to the minimum dimension of printed silver ink traces, only limited by the printer spatial resolution. The right inset of Figure 1 illustrates one of the eight types of antennas with the notation of geometrical parameters. The full sample area consisted of 30×30 V-shaped antennas, with its central frequency expected to be around 0.25 THz.

All samples were printed using a Ceradrop F-Serie Inkjet Printer (Limoges, France) with 1 pl Dimatix cartridge (FUJIFILM Dimatix, Santa Clara, CA, USA) that dispensed drops with a droplet spacing (center-to-center distance between ejected drops) of 20 μm. Only one nozzle was used to perform the printing. The jetting frequency was set at 500 Hz. We used a commercially available conductive silver ink DGP 40TE-20C (ANP, Pleasanton, CA, USA) that contains silver nanoparticles (Ag NPs) of sizes around 50 nm with 30–35 wt.% in triethylene glycol monomethyl ether solvent [39]. The substrate used for printing was a heat-stabilized polyethylene terephthalate (PET) polyester film (Melinex ST505, New Berlin, WI, USA). The chuck was maintained at a constant temperature of $60{\;}^{\circ }$C during the printing process. An in situ Adphos Near Infrared (NIR) Dryer Module CER-42-250 (Bruckmühl, Germany) was used to perform the annealing step of the printed patterns. The displacement time of the lamp was varied from 0.03 s/mm to 0.2 s/mm in order to obtain a set of samples with different thermal histories, resulting in a range of conductivities. A confocal laser microscope (Olympus LEXT OLS4000, Center Valley, PA, USA) was used to determine the thickness of the printed structure, which was found to be around 400 nm. The precise definition of the V-shaped antennas observed in the left inset of Figure 1 confirms the ability of the inkjet printer to achieve the proper design.

### 2.2. THz Time-Domain Spectroscopy (THz-TDS)

Assessments of the VPP control pattern were performed using THz-TDS measurements. An ultrafast Ti:Sapphire oscillator laser with a center wavelength of 805 nm producing pulses with a duration of ∼20 fs and a repetition rate of 80 MHz was used in combination with two LT-GaAs photoconductive antennas from Teravil (Vilnius, Lithuania). A beam splitter 80:20 splits the laser beam into an optical pump and a probe beam for THz generation and detection, respectively. The emitter and the detector were placed in front of each other, separated by a distance of around 40 cm. An optical chopper at 330 Hz was placed just after the emitter, allowing for lock-in detection. The samples were placed between the emitter and detector at normal incidence for transmission spectroscopy in air at room temperature and pressure.

**Figure 2:**
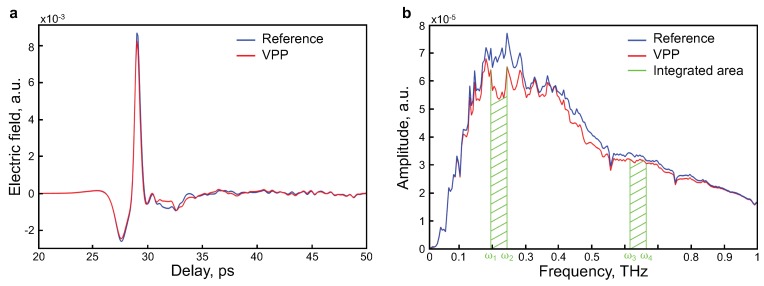
(**a**) time-domain spectra of VPP and its reference; (**b**) transmitted amplitude THz spectra of a VPP and a substrate. Representation of dual-wavelength THz spectroscopy (DWTS) principle: with transmitted amplitude spectrum of the VPP and a substrate, where we can see the transmission dip from $\omega_{1}$ to $\omega_{2}$ and no difference from $\omega_{3}$ to $\omega_{4}$.

To obtain the THz transmission value of the VPP sample, two THz pulses were acquired in the time domain, i.e., the reference ($E_{ref}\left(t\right)$) and the sample ($E_{sam}\left(t\right)$) signals, as shown in Figure 2a. An unpatterned PET substrate served as a reference. The normalized transmission T(ω) was obtained in the frequency domain using the following relation [40]:

(1) $$T(\omega )=|\frac{E_{ref}\left(\omega \right)}{E_{sam}\left(\omega \right)}|.$$

The vortex beam retained its shape after propagating through a homogeneous medium or at the focus of a lens [41]. This point is crucial in order to still be able to retrieve the transmission dip at vortex frequency using a single pixel detector (i.e., at the focus of a photoconductive THz detector).

### 2.3. Dual-Wavelength THz Spectroscopy (DWTS)

The analysis of THz-TDS data via normalized amplitude in the frequency domain required two THz measurements: reference and signal, respectively. Unfortunately, these measurements are sensitive to environmental conditions, which could induce some unwanted variations between each subsequent measurement. For spectroscopic methods in the visible and ultraviolet range, such unwanted fluctuations are often avoided by a dual wavelength measurement approach. The principle is simple: simultaneously measuring at two wavelengths (reference and signal) and recording the difference values at these wavelengths, also called balanced measurement [42,43,44,45]. This method has been used in the medical field to extract the concentration of drugs in tablets using UV radiation [43,44]. The idea of such methods is to find an intensity dependence ratio between the active element (signal) and the matrix (reference). After a proper calibration, this value is directly proportional to the concentration of an element of interest.

Conventional photoconductive THz antennas emit THz radiation that covers a broad range of frequencies, e.g., typically from 100 GHz to 10 THz. Therefore, differentiating between two distinct signal frequencies, within the same pulse spectrum, is a straightforward manipulation. As shown in Figure 2b, the process involves the extraction of a signal defined by a specific and narrowband range of frequencies, i.e., from $\omega_{1}$ to $\omega_{2}$, which exhibits a distinctive response proportional to the desired parameter (e.g., conductivity). A second frequency range, from $\omega_{3}$ to $\omega_{4}$, where no sign of absorption from the sample is detected, is used as reference information. The ratio between these two zones provides information about a transmission level corresponding to the parameter behavior under investigation. Since both signals are taken simultaneously, the noise from the ambient condition is suppressed in the normalization process:

(2) $$I=\frac{\int_{\omega_{1}}^{\omega_{2}}\left|E_{sam}\left(\omega \right)\right|d\omega }{\int_{\omega_{3}}^{\omega_{4}}\left|E_{sam}\left(\omega \right)\right|d\omega },$$

where *I* is the value of ratio and $|E_{sam}\left(\omega \right)|$ is the amplitude signal of the THz spectrum.

### 2.4. Comparison with Conventional Techniques

To validate the viability of characterizing printed electronics by electromagnetic THz waves, we employed two conventional conductivity measurement techniques: a multimeter with two probes and the state-of-the-art four-point probe methods. In addition, we performed AFM measurements of the surface morphology. Using a conventional multimeter instrument (MM) and two microprobes (S-shaped tungsten micro-probe tips), the electrical conductivity of a print pattern can be extracted using the following equation:

(3) $$\sigma =\frac{L}{RA_{c}},$$

where σ is the electrical conductivity, *R* is measured resistance, *L* and $A_{c}$ are the length and the cross-section area of a tested printed structure, respectively [13].

For higher precision, the four-point probe method (4PP) enables precise measurements of the electrical conductivity for a tiny sample within the area of the 4PP arrangement. To ensure a perfect match between our sample size and the 4PP tips, the spacing between probes was set to 100 μm (MCW-28-7188, GGB industries, Naples, FL, USA ). The measurement with 4PP provides a sheet resistance in which the conductivity value is extracted using the following equation [46]:

(4) $$\sigma =\frac{\ln2}{\pi tR},$$

where the geometric factor ln2/π describes the current rings emanating from the outer probe tips [46], *t* is the thickness of the patch and *R* is the measured sheet resistance.

To confirm the good agreement between the conductivity of the printed control bar and the conductivity value of the patch, we also measured the resistance of a V-shaped antenna with two microprobes (2MP) and extracted its conductivity using Equation (3). Finally, to ensure that the sintering speed was responsible for the changes in conductivity, the surface morphologies of the printed samples were characterized using the AFM (EnviroScope, Santa Barbara, CA, USA) system in tapping mode.

## 3. Results and Discussion

**Figure 3:**
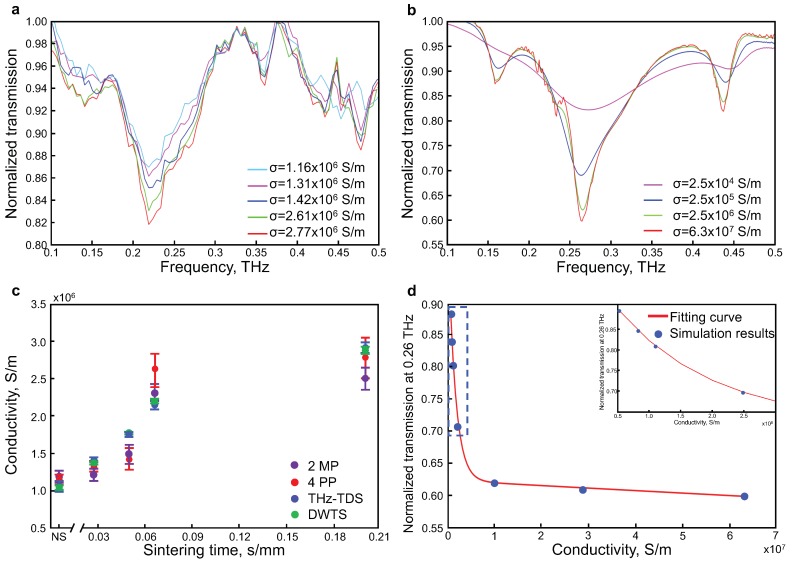
(**a**) normalized transmission spectra of experimental results; (**b**) simulated normalized transmission spectra with finite difference time domain (FDTD) method; (**c**) comparison of the conductivity values obtained by two microprobes (2MP) (violet), 4PP (red), normalized transmission from THz-TDS (blue) and DWTS (green) as a function of sintering time; (**d**) simulated transmission amplitude at 0.26 THz vs. conductivity.

Five VPP samples with different conductivities were characterized by the THz-TDS described above. The conductivity of each sample was controlled by varying the sintering time. One of the samples (non-sintered) was not sintered by the lamp, but was slightly sintered during the printing step, since the chuck was held at a constant temperature of 60 ${}^{\circ }$C. Figure 3a illustrates the normalized transmission amplitude of the different VPP samples, which were obtained from Equation (1). A dip in the transmission is observed due to the generation of a vortex beam at 0.22 THz, as expected [37]. As mentioned previously, a higher resonance response (i.e., which translates to a lower transmission at 0.22 THz) indicates a sample with higher electrical conductivity.

To validate the accuracy of THz sensing of vortex plates as a function of material conductivity, we performed finite difference time domain (FDTD) simulations using the Lumerical software. Linearly polarized waves and perfectly matched layer boundary conditions were used in the simulation.

Figure 3b shows the simulated transmission spectra of VPPs with defined and uniform conductivities of a hypothetical printed metal. We placed VPP in the air in order to avoid Fabry–Perot resonances from the substrate. We can observe three transmission dips; the strongest one at 0.265 THz represents the central frequency of VPP. Compared to experiments, the red shift of the central frequency is explained by the absence of the PET substrate.

The simulation and experiment differ in the degree of transmission difference as a function of metal conductivity. We attribute this difference to the perfect reading of the central vortex information in the simulated case. Essentially, the photoconductive antenna reads a spatially integrated range of information containing the central intensity part of a donut shaped beam, together with a large contribution from its wings. Nevertheless, the numerical simulations are in good agreement with experimental findings.

Figure 3c gives the measured conductivity of five samples using three different methods: 2MP, 4PP, THz-TDS and DWTS as a function of sintering speed. The 4PP method was performed on the patch samples, while 2MP, THz-TDS and DWTS measurements provide the corresponding conductivity results from the VPP samples. The function of the value of the dip in transmission against the conductivity of VPP was also simulated, as shown in Figure 3d. It is important to note that this function clearly reveals the extremely high sensitivity of THz wave sensing for low conductivity samples (e.g., below $1\times 10^{7}$ S/m, the blue dotted region in inset). Above this conductivity value, the dip in transmission exhibits less sensitivity, with an almost saturated behavior (i.e., closer to a perfect metal resonance).

To compare the performance of THz-TDS and 4PP, the THz transmission amplitudes at 0.22 THz were calibrated to the expected conductivity values obtained from 4PP. Since the 4PP measurements cover a limited range of conductivity, from $1\times 10^{6}$ to $3\times 10^{6}$ S/m, a simple calibration using a linear fit was chosen (in agreement with inset of Figure 3d, with the non-sintered sample as the starting point. In Figure 3c, the similar increases in conductivity behavior as a function of sintering exposure time for the measurements taken by THz-TDS and 4PP is observed. More importantly, all sintering conditions are well discriminated by THz measurements, whereas 4PP failed in differentiating the three lowest conductivity conditions (i.e., <$1.5\times 10^{6}$ S/m), as well as the two highest conductivity conditions (i.e., >$2.5\times 10^{6}$ S/m). In addition, we repeated the measurements ten times for each method and calculated the standard deviation. Interestingly, THz-TDS exhibits better repeatability than the conventional 4PP method. We attribute this difference to the contactless nature of the THz method: 4PP can locally damage the ink surface and may render repeated measurement less accurate.

In the second step, using the data obtained from THz-TDS measurements, we analyzed the sample signal by the DWTS method. The two frequency ranges were 0.195–0.244 THz and 0.615–0.664 THz, for the signal and reference, respectively (see Figure 2b). In order to perform the measurement in ambient conditions, the reference frequency range was chosen to avoid the water absorption lines that can occur due to ambient humidity. Similarly to THz-TDS transmission data, the integral values from DWTS were normalized and calibrated with respect to the retrieved conductivity using the 4PP method. The behavior follows the expected static conductivity, but more importantly, the repeatability is four times better than the conventional 4PP method.

In the final step, we review the analysis done on the patch versus VPP samples using the various methods described previously. Table 1 summarizes the obtained results. In order to establish a comparative measurement performance, we carried out several resistance measurements at different locations for the patch and V-shaped antenna and present their relative standard deviation (RSD). As mentioned previously, the 4PP and multimeter retrieved the resistance on the patch. To clearly validate that VPP conductivity is linked to the patch conductivities, 2MP were also used to evaluate the VPP resistance directly. It has to be mentioned that, due to the extremely small effective volume of VPP unit cell, the 2MP method can easily over- or underestimate the conductivity (e.g., conductivity dependency on sample volume, as shown in Equation (3)). However, the 2MP measurements confirmed the good agreement between the sintering exposure time for the patch and VPP samples together.

In order to confirm the provided conductivity measurements, the evolution of the sintering of Ag ink was studied using AFM analysis at five different sintering stages. The last row of Table 1 depicts the printed ink surfaces after sintering. The non-NIR-sintered sample (NS) showed poor contact between Ag NPs, resulting in the lowest conductivity ($1.15\times 10^{6}$ S/m). The sample with the shortest annealing time (0.03 s/mm) depicted the next stage of the sintering, necks began to grow between NPs prompted by surface energy minimization. With a longer annealing time of 0.05 s/mm, the NPs get more compact and the printed structure densifies. The slight increase of annealing time to 0.07 s/mm led to a further increase in conductivity. The longest annealing time (0.2 s/mm) led to the highest density and the highest conductivity ($2.77\times 10^{6}$ S/m). According to AFM observations of the surface morphology of the samples, the obtained samples were consistent with the sintering parameters and measurements of the conductivity with different techniques.

As can also be seen in Table 1, as expected, the measurements provided by a conventional multimeter were the least precise since the probes of the multimeter easily break the surface of the patch after contact. Meanwhile, the micro-probe provides a safer way to avoid destroying the sample surface. The average conductivities measured with the different techniques are in the same range, and have similar behavior as a function of the sintering time. It should be emphasized that the trend in electrical static conductivity measurements on the printed patch and the VPP using the different techniques are all in good agreement. This confirms the feasibility of characterizing the variability in ink conductivity during mass production of PE devices simply by reading a test structure. Finally, the best RSD for repeatability was obtained for DWTS and THz-TDS.

## 4. Conclusions

In conclusion, we developed a quality control bar for industrial production of PE devices based on a VPP working in the THz range. The VPP was formed from V-shaped antennas with a central frequency at 0.220 THz. The samples were printed with commercially available ink consisting of silver nanoparticles, and a commercial inkjet printer was used for the fabrication. The conductivities of the printed samples were varied by changing the speed of a near-infrared heater. THz-TDS was employed to analyze the transmission properties of printed VPP. Our results showed that the THz transmission response of a resonant sample enables to follow the changes in sintering condition of the printed ink. We validated our results with a simulation study and introduced DWTS as a simple and fast method to quickly determine the transmission response of VPP. Our analysis also confirms the similar conductivity behavior between adjacent printed structures and VPP sample as function of sintering exposure time. This important observation enables to track the changes in sintering process of PE devices during the manufacturing process using a simple control bar.

Finally, using the conventional four-point-probe method as a reference, we confirmed that a calibrated quality control bar in the shape of the VPP could be used to determine the static electrical properties of non-resonant printed devices that are printed simultaneously with the VPP samples. Being a non-contact method, it is highly suitable for in-line characterization of high-speed roll-to-roll printing repeatability of PE devices. 

## Figures and Tables

**Table 1:** Comparison between terahertz time-domain spectroscopy (THz-TDS) and conventional techniques. The scale of atomic force microscopy (AFM) images is the same for all figures.

Sintering Time			Not Sintered	0.03 s/mm	0.05 s/mm	0.07 s/mm	0.2 s/mm
Conductivity, $\times 10^{6}$ S/m	Obtained by	4 PP	1.15 ± 4.70%	1.31 ± 4.47%	1.42 ± 10.3%	2.61 ± 8.67%	2.77 ± 9.82%
		MM	1.10 ± 21.1%	1.19 ± 12.1%	1.48 ± 17.9%	2.15 ± 22.2%	3.01 ± 36.7%
		2MP	1.23 ± 6.59%	1.28 ± 8.44%	1.49 ± 8.73%	2.29 ± 6.83%	2.51 ± 7.58%
		THz-TDS	1.08 ± 6.14%	1.38 ± 3.06%	1.75 ± 1.55%	2.14 ± 2.52%	2.91 ± 2.77%
		DWTS	1.06 ± 3.44%	1.37 ± 1.28%	1.78 ± 1.21%	2.18 ± 1.44%	2.89 ± 1.80%
AFM			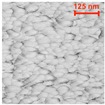	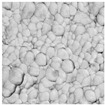	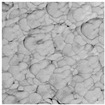	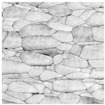	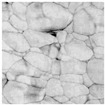

4PP—four-point probe; MM—multimeter; 2MP—two microprobes; THz-TDS—terahertz time-domain spectroscopy; DWTS—dual-wavelength terahertz spectroscopy; AFM—atomic force microscopy.
